# Comparison of target agent treatment strategies for platinum-resistant recurrent ovarian cancer: A Bayesian network meta-analysis

**DOI:** 10.1097/MD.0000000000038183

**Published:** 2024-05-24

**Authors:** John Hang Leung, Henry W. C. Leung, Shyh-Yau Wang, Hei-Tung Yip Fion, Agnes L. F. Chan

**Affiliations:** a Department of Obstetrics and Gynecology, Ditmanson Medical Foundation Chia-Yi Christian Hospital, Chia-Yi, Taiwan; b Department of Radiation Oncology, An-Nan Hospital, China Medical University, Tainan, Taiwan; c Department of Radiation, An-Nan Hospital, China Medical University, Tainan, Taiwan; d Department Management Office for Health Data, Clinical Trial Research Center, China Medical University Hospital, Taichung, Taiwan; e Department of Pharmacy, An-Nan Hospital, China Medical University, Tainan, Taiwan.

**Keywords:** anti-angiogenic agents, immune checkpoint inhibitors, network meta-analysis, platinum-resistant recurrent ovarian cancer, target therapy

## Abstract

**Background:**

We aimed to compare 7 newer immunotherapies and targeted therapies for platinum-resistant relapsed ovarian cancer.

**Methods:**

We conducted a comprehensive search of PubMed, EMBASE, and Cochrane Library electronic databases for phase III trials involving platinum-resistant recurrent ovarian cancer (PRrOC) patients treated with immunotherapy or targeted therapy in combination with chemotherapy. The quality of the included trials was assessed using the GRADE method. The primary outcome of comparison was progression-free survival, and secondary outcomes included overall survival and safety.

**Results:**

This analysis included 7 randomized phase III controlled trials, encompassing 2485 PRrOC patients. Combining bevacizumab plus chemotherapy and lurbinectedin demonstrated statistically significant differences in progression-free survival compared to all other regimens of interest. However, no statistically significant differences were observed in the overall survival. Nivolumab and mirvetuximab exhibited fewer serious adverse events than the other regimens of interest.

**Conclusions:**

Our findings indicate that bevacizumab combined with chemotherapy and lurbinectedin monotherapy has significant efficacy in patients with PRrOC. For patients with PRrOC who have exhausted treatment options, nivolumab and mirvetuximab may be considered as alternatives because of their better safety profiles.

## 1. Introduction

In 2020, ovarian cancer (OC) had an incidence and mortality of 308,102 and 207,252 cases, respectively, making it the fifth leading cause of cancer-related deaths among women worldwide.^[1]^ In Taiwan, OC was ranked as the tenth leading cause of cancer-related deaths in the same year.^[2]^ Survival rates depend heavily on the disease stage at diagnosis. Most patients are diagnosed at an advanced stage III to IV, resulting in a 5-year survival rate of approximately 40% to 45%.^[3]^ The lack of effective screening methods and absence of specific symptoms contribute to delays in disease diagnosis.^[4]^

Debulking surgery and platinum-based chemotherapy are considered the first-line standard of care for newly diagnosed OC.^[5]^ However, despite initial high response rates, most patients experience relapse within 3 years.^[6]^ Approximately 20% to 25% of patients develop platinum-resistant or platinum-refractory disease, resulting in a poor prognosis with a median overall survival (OS) of <12 months.^[7,8]^ Consequently, there is an urgent need for more effective treatment strategies.

The treatment of platinum-resistant ovarian cancer (PRrOC) remains a significant unmet need in the gynecologic oncology community. Numerous molecular-targeted drugs, including inhibitors of signaling pathways, anti-angiogenic agents, poly(ADP-ribose) polymerase (PARP) inhibitors, selective estrogen receptor down regulators, immune checkpoint inhibitors, and various chemotherapy combinations, have been investigated to improve outcomes in PRrOC. However, the results have been discouraging, showing limited activity regarding radiological/biochemical response and progression delay without any therapy demonstrating a clear improvement in OS.^[8]^ In recent years, the combination of bevacizumab plus chemotherapy in the “AURELIA” study demonstrated statistically significant improvements in progression-free survival (PFS) and overall response rate, but did not show a benefit in terms of OS.^[9]^ Moreover, 2 new targeted agents, mirvetuximab and lurbinectedin, have recently been approved for the treatment of PRrOC based on the results of the FORWARD and CORAIL phase III randomized controlled trials. Mirvetuximab is a molecularly targeted antibody-drug conjugate that specifically targets folate receptor alpha (FRα), a cell surface protein that is heterogeneously overexpressed in epithelial OC. Conversely, lurbinectedin is a DNA minor groove covalent binder with potent antitumor activity that preferentially binds to guanines located within the GC-rich regulatory regions of DNA gene promoters. Additionally, lurbinectedin has demonstrated the ability to modulate the tumor microenvironment and exhibits activity against cancer cells with deficiencies in homologous recombination DNA repair.^[10,11]^ Given the lack of direct head-to-head evidence, we aimed to evaluate and rank the efficacy of these new treatment options for PRrOC using a Bayesian network meta-analysis. The results of this study could serve as an updated reference for gynecologists and healthcare decision-makers in selecting effective and safe therapeutic strategies for patients with PRrOC.

## 2. Methods

### 2.1. Search strategy and study selection

We conducted a comprehensive literature search for eligible randomized controlled trials (RCTs), limited to English, using PubMed, Embase, and Cochrane Central Register of Controlled Trials databases. The search included articles published between January 2014 and December 2021 following the Preferred Reporting Items for Systematic Reviews and Meta-Analyses (PRISMA) guidelines.^[12]^ The detailed search strategies are described in the Supplementary Information (available online, http://links.lww.com/MD/M559). Additionally, we manually searched for additional references to identify potentially overlooked studies.

All included trials met the following criteria: randomized phase III clinical trials comparing chemotherapy with a combination of immunotherapeutic agents (nivolumab and avelumab) or targeted agents (bevacizumab, pertuzumab, mirvetuximab, and lurbinectedin) with chemotherapeutic agents in patients with PRrOC, defined as having a progression-free interval (PFI) of <6 months; reporting severe adverse events (SAEs) above grade 3; and reporting efficacy outcomes as PFS and OS. If the updated trials were published, only the latest results were included in the analysis. Another author examined the final selected trials to verify their adherence to the inclusion criteria. Two independent authors (LH and AC) assessed the data extracted from the eligible RCTs. A third reviewer (LJ) was consulted for any controversy and to reach a consensus.

The quality of the selected RCTs was assessed using the Cochrane Collaboration risk-of-bias assessment method, which included the evaluation of selection bias, performance bias, detection bias, attrition bias, reporting bias, and other biases. Each domain was explicitly assessed as having a low, high, or unclear risk-of-bias (due to either a lack of information or uncertainty regarding bias).^[13]^

### 2.2. Outcomes of interest

The primary outcome was PFS, defined as the time from randomization to disease progression (according to the RECIST v1.1 criteria) or death. Secondary outcomes included overall survival (OS), defined as the time from randomization to death from any cause. SAEs were classified as grade 3 or higher according to the National Cancer Society Common Terminology Criteria for Adverse Events, Version 5.0.^[14]^

The cumulative ranking probabilities for the 9 treatment regimens (bevacizumab + chemotherapy, nivolumab, pertuzumab + chemotherapy, avelumab + chemotherapy, mirvetuximab, lurbinectedin, phenoxodiol (PXD) + Carboplatin, avelumab, chemotherapy) were calculated using the surface under the cumulative ranking (SUCRA) curve. The SUCRA values were derived from the distribution of ranking probabilities. Higher SUCRA numbers indicate the most effective treatment regimens in the network meta-analysis. The ranking of SAEs for each regimen is presented as a heatmap, which was generated based on the rankogram data from Table 1.

**Table 1:** The league table of comparisons for severe ADE.

Nivolumab								
0.15 (0.07–0.32)	Mirvetuximab							
0.14 (0.06–0.31)	0.94 (0.47–1.92)	Avelumab						
0.13 (0.06–0.25)	0.84 (0.46–1.55)	0.90 (0.47–1.67)	Lurbinectedin					
0.09 (0.03–0.22)	0.58 (0.25–1.37)	0.61 (0.26–1.47)	0.69 (0.30–1.57)	Pert + chemo				
0.06 (0.03–0.11)	0.42 (0.26–0.68)	0.45 (0.27–0.74)	0.50 (0.34–0.74)	0.73 (0.35–1.49)	Chemo			
0.04 (0.01–0.12)	0.27 (0.10–0.73)	0.28 (0.10–0.78)	0.32 (0.12–0.84)	0.46 (0.14–1.47)	0.63 (0.25–1.53)	PXD + Carbo		
0.04 (0.02–0.08)	0.25 (0.13–0.47)	0.26 (0.14–0.50)	0.29 (0.16–0.53)	0.43 (0.18–0.99)	0.59 (0.38–0.89)	0.93 (0.34–2.49)	Avelumab + chem	
0.02 (0.01–0.06)	0.15 (0.06–0.35)	0.16 (0.07–0.37)	0.18 (0.08–0.39)	0.26 (0.09–0.70)	0.36 (0.17–0.70)	0.56 (0.18–1.73)	0.60 (0.26–1.40)	Bev + chemo

Data are presented as odds radio (OR) and 95% confidence intervals (CI). OR < 1 favors the treatment on top left.

chemo = chemotherapy, PXD = phenoxodiol.

### 2.3. Statistical analysis

Traditional pairwise meta-analyses were conducted using Review Manager Software version 5.4 to estimate pooled effect sizes with odds ratios (ORs) as the measurement. Heterogeneity was assessed using the *I*^2^ test, considering *I*^2^ values > 50% as indicative of heterogeneity. The choice between a random-effects model and a fixed-effects model depended on the presence or absence of statistical heterogeneity, and a random-effects model was selected if significant heterogeneity was observed. The results are reported as ORs with corresponding 95% confidence intervals (CIs). Statistical significance was defined as *P* < .05.

To compare the efficacy and safety of 7 targeted therapies and immunotherapies for PRrOC, Bayesian Markov Chain Monte Carlo method in WinBUGS 1.4.3 (MRC Biostatistics Unit, Cambridge, and Imperial College School of Medicine, London, UK) and NetMetaXL (version 1.6.1) were utilized.^[15]^ The choice between a fixed-effects model and a random-effects model was determined based on the deviance information criteria (DIC) and the convergence of the model. Model convergence was assessed using the Brooks-Gelman-Rubin method, which compares within-chain and between-chain variances to calculate the potential scale-reduction factor. Convergence is achieved when the value is close to 1.0.^[16]^ Inconsistencies between direct and indirect evidence were assessed by plotting the posterior mean deviation for individual data points in the inconsistency model against the posterior mean deviation in the consistent model to identify potential inconsistencies within the network. Sensitivity analyses were performed using both fixed-effects and random-effects models to test the robustness of the network comparisons by repeating the main calculations.

## 3. Results

### 3.1. Study search and characteristics of the included RCTs

A total of 53 relevant articles were identified by searching the PubMed, EMBASE, and Cochrane Library electronic databases (as of December 2021) (Supplementary Figure 1, http://links.lww.com/MD/M556). Seven randomized, double-blind, phase III controlled trials met the inclusion criteria. The analyses included a total of 2485 PRrOC patients.^[10,11,17–21]^ The median age of the patients ranged from 25 to 89 years across all the studies. All RCT compared the investigated combination of targeted therapies and immunotherapies with chemotherapy. The years of publication of the included studies ranged from 2014 to 2021. Five studies were phase III open-label RCTs^[10,11,18,19,21]^ and 2 were double-blinded RCTs.^[17,20]^ The characteristics of the 7 studies are presented in Table 2. The quality of the evidence was assessed using the Cochrane Risk-of-Bias tool.^[13]^ The risk-of-bias was low for most categories. Five phase III studies were open-label and unblinded to the participants and outcome assessors, resulting in a high risk of performance bias and a median risk of selection and detection bias. The results of the quality assessment are shown in Supplementary Figure 2, http://links.lww.com/MD/M557.

**Table 2:** Study characteristics of trials included in the network meta-analysis.

Author/yr	Design	Population	No. of patients	Median age yr (range)	Intervention group	Control group	Outcomes (mo)				
							Patient number/Survival mo				
								Median P FS	N	Median OS	ORR(N)
Lauraine 2014^[9]^ AURELIA	Phase III Open-label	Platinum ROC	179 182	62 (25–80) 61 (25–84)	Bevacizumab + Chemo	Chemotherapy	135 166	6.7 3.4	128 136	16.6 13.3	55 33
Lauraine 2021^[18]^ JAVELIN Lauraine -1 2021	Phase III Open-label	Platinum ROC	188 190 188	60 (53–67) 60 (53–69) 61 (63–69.5)	Avelumab + Chemo Avelumab	Chemotherapy	134 125 154	3.7 3.5 1.9	102 104 109	15.7 13.1 11.8	25 8 7
Kurzedev 2016^[17]^ PENELOPE	Phase III Double-blinded	Platinum ROC	78 78	65 (32–79) 64 (26–80)	Pertuzumab + Chemo	Chemotherapy	66 60	4.3 2.6	39 43	10.3 7.9	9/61 6/69
Hamanishi 2021^[19]^ NIVJA	Phase III Open-label	Platinum ROC	157 159	<65 >65	Nivolumab	Chemotherapy	83 84	2.0 3.8	131 125	10.1 12.1	9/119 15/114
Moore, 2021^[10]^ (FORWARD-1)	Phase III Open-label	Platinum RROC	248 118	64 (34–89) 64 (31–86)	Mirvetuximab	Chemotherapy	174 70	10 8.4	96 50	16.4 14	55 14
Gaillard 2021^[21]^ CORAIL	Phase III Open-label	Platinum RROC	221 221	63 (25–85) 59 (28–87)	Lurbinectedin	Chemotherapy	179 158	3.5 3.6	170 168	11.4 10.9	32 28
Fotopoulou 2014^[20]^	Phase III Double-blinded	Platinum RROC	70 72	57.5 (39–78) 59 (37–82)	PXD + Carboplatin	Chemotherapy	33 34	3.8 5.0	35 36	9.6 11.4	1 0

ORR = objective response rate, OS = overall survival, PFS = free survival, ROC = resistant-ovary cancer, chemotherapy for AURELIA: pegylated liposomal doxorubicin, palitaxel, topotecan; chemotherapy for JAVELIN：pegylated liposomal doxorubicin; chemotherapy for PENELOPE: palitaxel, topotecan, gemcitabine; chemotherapy for NIVIA: pegylated liposomal doxorubicin or gemcitabine; chemotherapy (Moore): paclitaxel, pegylated liposomal doxorubicin, or topotecan; chemotherapy for CORAIL: PLD or topotecan; PXD: phenoxodiol.

### 3.2. Bayesian network meta-analysis

First, we assessed the goodness of fit for the fixed- or random-effects model based on the DIC. The DIC values for PFS and OS were 0.075 and 0.1467, respectively, with differences of <2.^[22]^ The Brooks-Gelman-Rubin test demonstrated better convergence for the fixed-effects model. Consequently, we employed a fixed-effects model for the NMA.

In terms of PFS, we observed significant improvements with bevacizumab plus chemotherapy and lurbinectedin compared to chemotherapy [(0.29; 0.15–0.53); (0.59; 0.38–0.92)], avelumab + chemotherapy [(0.22; 0.09–0.53); (0.46; 0.25–0.87)], pertuximab + chemotherapy [(0.17; 0.06–0.48); (0.36; 0.14–0.89)], and mirvetuximab[(0.18; 0.08–0.39); (0.37; 0.19–0.70)]. Additionally, bevacizumab plus chemotherapy showed a more favorable improvement in PFS compared to nivolumab (0.31; 0.14–0.67) and pxd + carboplatin (0.29; 0.11–0.29). However, avelumab was slightly inferior to bevacizumab plus chemotherapy (0.12; 0.05–0.26), lurbinectedin (0.25; 0.13–0.48), nivolumab (0.39; 0.20–0.76), pxd + carboplatin (0.42; 0.26–0.68), and chemotherapy alone (0.42; 0.18–0.94), but there was no significant difference with other regimens of interest. Regarding OS, no significant improvement was observed with any of the regimens of interest compared with chemotherapy alone (Table 3).

**Table 3:** The league table for comparisons of progression survival (PFS) and overall survival (OS).

Bev + chemo	0.78 (0.42–1.48)	0.61 (0.29–1.27)	0.84 (0.53–1.34)	0.84 (0.38–1.90)	0.86 (0.46–1.60)	1.02 (0.46–2.25)	0.97 (0.52–1.86)	0.73 (0.40–1.36)
0.49 (0.22–1.05)	Lurbinectedin	0.79 (0.37–1.62)	0.93 (0.60–1.46)	0.92 (0.42–2.04)	0.91 (0.50–1.67)	0.76 (0.35–1.65)	0.79 (0.43–1.52)	0.94 (0.52–1.72)
0.31 (0.14–0.67)	0.64 (0.34–1.20)	Nivolumab	0.73 (0.41–1.28)	0.73 (0.30–1.75)	0.71 (0.35–1.43)	0.59 (0.25–1.40)	0.62 (0.30–1.27)	0.83 (0.40–1.67)
0.29 (0.15–0.53)	0.59 (0.38–0.92)	0.93 (0.59–1.45)	Chemo	0.99 (0.52–1.92)	0.98 (0.65–1.47)	0.82 (0.43–1.57)	0.86 (0.55–1.35)	0.73 (0.41–1.28)
0.29 (0.11–0.72)	0.59 (0.27–1.32)	0.93 (0.41–2.08)	1.00 (0.52–1.95)	PXD + Carboplatin	0.99 (0.46–2.11)	0.83 (0.33–2.07)	0.87 (0.39–1.90)	0.87 (0.40–1.88)
0.22 (0.09–0.53)	0.46 (0.25–0.87)	0.72 (0.39–1.36)	0.77 (0.50–1.21)	0.78 (0.35–1.70)	Avelumab + chem	0.83 (0.40–1.78)	0.88 (0.48–1.60)	0.71 (0.35–1.43)
0.17 (0.06–0.48)	0.36 (0.14–0.89)	0.56 (0.22–1.40)	0.60 (0.26–1.34)	0.60 (0.21–1.72)	0.77 (0.30–1.93)	Pert + chemo	0.95 (0.44–2.08)	0.72 (0.34–1.54)
0.18 (0.08–0.39)	0.37 (0.19–0.70)	0.58 (0.31–1.10)	0.62 (0.40–0.97)	0.62 (0.28–1.39)	0.80 (0.42–1.48)	1.03 (0.41–2.68)	Mirvetuximab	0.75 (0.41–1.40)
0.12 (0.05–0.26)	0.25 (0.13–0.48)	0.39 (0.20–0.76)	0.42 (0.26–0.68)	0.42 (0.18–0.94)	0.54 (0.28–1.05)	0.71 (0.27–1.84)	0.68 (0.35–1.31)	Avelumab

Data are presented as odds radio (OR) and 95% confidence intervals (CI). For PFS, OR < 1 favors the treatment on left column; For OS, OR < 1 favors the treatment on left row. Yellow color presented PFS and green color was OS.

chemo = chemotherapy.

**Figure 1.**
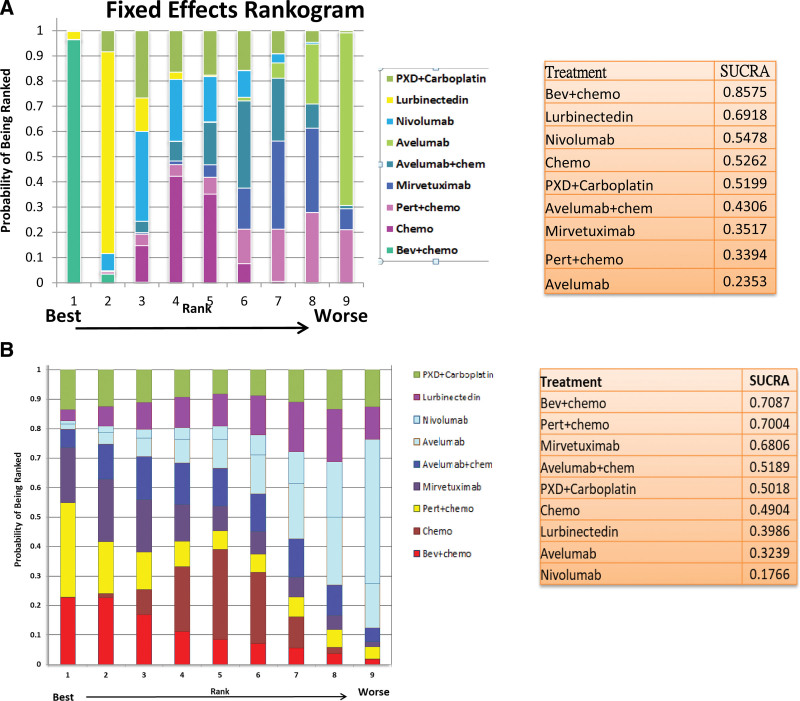
Ranogram and SUCRA ranking for PRrOC (A) PFS (B) Overall survival. PRrOC = platinum-resistant recurrent ovarian cancer, SUCRA = surface under the cumulative ranking.

We used the SUCRA curve to determine the rank probability of PFS and OS for each treatment regimen. Bevacizumab plus chemotherapy showed the highest rank in terms of PFS improvement, followed by lurbinectedin and nivolumab. Conversely, avelumab ranked the lowest in terms of efficacy. Regarding OS, bevacizumab plus chemotherapy achieved a higher rank, whereas nivolumab achieved a lower rank (Fig. 1).

**Figure 2.**
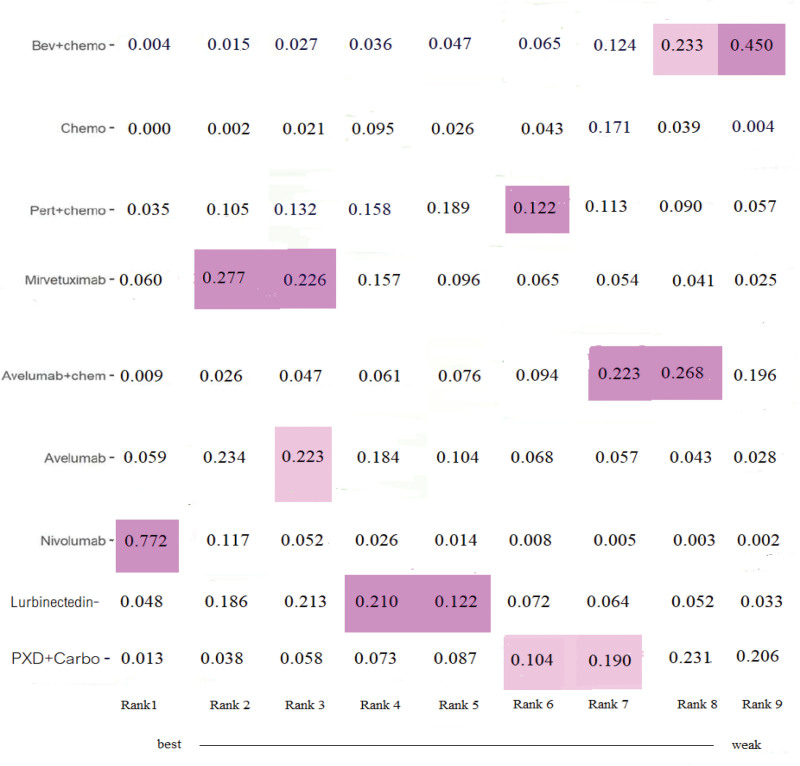
SAE heatmap ranking for severe adverse events. Bev (bevacizumab) + Chemo1 (pegylated liposomal doxorubicin, palitaxel, topotecan); PXD (Phenoxodiol) + carboplatin; Pert + chemo (pertuzumab + topotecan, weekly paclitaxel, or gemcitabine). chemo = chemotherapy, SAEs = severe adverse event.

A comparison of the SAEs is presented in the form of a heatmap plot. Nivolumab, mirvetuximab, and avelumab were the top 3 regimens with a favorable safety profile, while bevacizumab plus chemotherapy and avelumab plus chemotherapy were identified as the regimens with a relatively higher risk of SAEs. (Fig. 2, Table 1).

**Figure 3.**
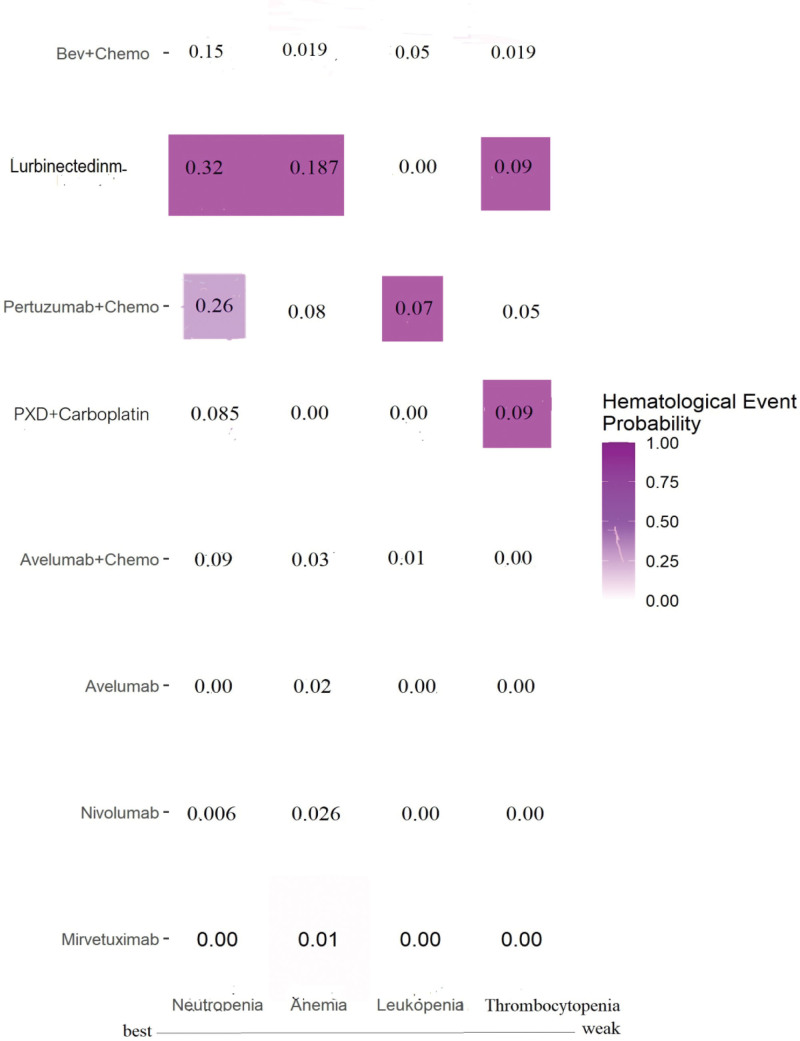
Hematologial SAE heatmap plot. SAEs = severe adverse event.

Subgroup analysis of severe hematologic SAEs, presented as a heatmap, revealed that severe neutropenia and anemia were more common with lurbinectedin, whereas thrombocytopenia and leukopenia were more frequently associated with lurbinectedin, pxd + carboplatin, and pertuzumab plus chemotherapy (Fig. 3). The most common non-hematological SAEs caused by bevacizumab plus chemotherapy were hypertension (7.2%) and hand-foot syndrome (5%). Pertuzumab plus chemotherapy and lurbinectedin induced diarrhea (14.3%) and fatigue (7.3%). GI perforation and proteinuria (2%) were associated with bevacizumab plus chemotherapy.

### 3.3. Assessment of inconsistency and sensitivity analysis

The inconsistency plots demonstrated no inconsistency between the direct and indirect studies for both PFS and OS, as all included studies were phase III RCTs (Supplementary Figure 3 AB, http://links.lww.com/MD/M558). The data points that were better fitted by the inconsistency model relative to the network meta-analysis (NMA), were close to 1 for both the consistency and inconsistency models. Sensitivity analysis results for network comparisons indicated no significant difference between the random- and fixed-effects models for PFS (Tau = 0.1263; 95% CrI: 0.0388–0.416) and OS (Tau = 0.1301; 95% CrI: 0.040–0.415), both in terms of direct and indirect comparisons. The results of this study are robust.

## 4. Discussion

In this study, we conducted both indirect and direct network comparisons of available phase III clinical trials involving patients with PRrOC. Based on our indirect network comparison, the combination of bevacizumab and chemotherapy exhibited a favorable improvement in PFS, albeit with a higher incidence of SAEs than chemotherapy and other target agents of interest. This finding seems to be supported by recent evidence suggesting that the addition of anti-angiogenic drugs to cytotoxic chemotherapy can modulate the composition and function of tumor-infiltrating lymphocytes and myeloid cells, subsequently positively affecting patient prognosis.^[23]^

Notably, neoadjuvant platinum-based chemotherapy significantly alters the tumor microenvironment in epithelial OC, as highlighted by the results of immunogenicity analysis. This analysis revealed that archived tumor samples were enriched for oligoclonal expansion of natural killer cells and T cells, leading to enhanced cytolytic activity and infiltration.^[24,25]^ Furthermore, platinum-based drugs induce changes in the immune system and promote the intratumoral migration of T cells by upregulating pro-inflammatory chemokines.^[24]^

Other cytotoxic chemotherapy products, such as PEGylated liposomal doxorubicin (PLD) and anthracycline, may function as immunosensitizers for dendritic cells and CD8 + T cells, thereby enhancing the host immune response against cancer cells.^[26]^ Consequently, PLD was hypothesized to alleviate tumor-induced immunosuppression, promote immune effector cell activation, and eliminate immunosuppressive myeloid-derived suppressor cells.^[27]^ Because many OC patients exhibit primary or secondary resistance to chemotherapeutic drugs, some researchers have explored combining immune checkpoint inhibitors (ICIs) with cytotoxic chemotherapy to identify effective therapeutic strategies for improving the prognosis of OC patients.^[28,29]^ Pujade-Lauraine et al conducted a 3-arm randomized phase III study (JAVELIN 200 trial) comparing a PD-L1 monoclonal antibody (avelumab) with PLD or avelumab versus PLD alone for PRrOC. Unfortunately, neither avelumab nor the combination therapy was effective, as there was no significant improvement in PFS or OS compared with PLD.^[30]^ Subsequently, the NINJA trial reported less promising results for nivolumab, with no statistically significant improvements in PFS and OS.^[19]^ Our NMA findings align with these trials, showing no significant improvements in PFS and OS with nivolumab, avelumab combined with PLD, or avelumab monotherapy compared to chemotherapy (PLD and gemcitabine). Given the lack of significant benefit of adding ICIs to cytotoxic chemotherapy, PARP inhibitors, or anti-angiogenic drugs, and the fact that for many years, the treatment of recurrent OC after platinum-based chemotherapy was based solely on the PFI, the development of new drugs in this setting is an interesting and challenging issue.

Recently, a phase III randomized multicenter CORAIL study reported that lurbinectedin exhibited similar antitumor efficacy and better tolerance to SAEs compared to PLD or topotecan in patients with PRrOC.^[21]^ Lurbinectedin, a novel synthetic alkaloid, demonstrated antitumor activity against platinum-resistant OC in preclinical models by forming a covalent bond with the central guanine in a specific nucleoside triplet located in the minor groove of the DNA molecules. This interaction leads to the formation of lurbinectedin DNA adducts, inducing double-strand breaks in cancer cells and perturbations in the cell cycle. Additionally, lurbinectedin modulates the tumor microenvironment and can evoke anticancer immunity. The tumor microenvironment (TME) plays a crucial role in tumor growth and treatment. With an improved understanding of the TME concept and major prognostic factors such as highly immunogenic tissue-specific antigen expression and immune infiltration,^[31]^ immunotherapy has emerged as another strategy for modulating the immune microenvironment to overcome resistance to PRrOC therapies.^[32]^ The present study indicates that tumor mutational burden (TMB) serves as an important biomarker that represents the degree of tumor mutation and predicts prognosis and response to ICIs^[33,34]^ Subsequent studies have found that tumors expressing programmed cell death ligand 1 (PD-L1) exhibit resistance to ICIs because of immunophenotypic markers, including TMB, programmed cell death protein 1, and PD-L1,^[35,36]^ as well as homologous repair deficient and proficient (HRP) phenotypes and tumor-infiltrating lymphocytes, which are factors influencing the tumor microenvironment (TME).^[37–39]^ The TMB-high phenotype has been extensively documented to predict response to ICIs in solid tumors.^[40–42]^ Unfortunately, OC exhibits a low TMB phenotype and a limited response to ICIs.^[43,44]^

Mirvetuximab (MIRV) represents another approach to enhance drug delivery to tumors in patients with PRrOC by targeting multiple resistance mechanisms. MIRV is an antibody-drug conjugate (ADC) that specifically targets FRα, which is expressed in approximately 80% of OCs and is absent in normal cells.^[45]^ ADC is composed of an FRα-binding antibody and the potent tubulin-targeting agent DM4, a maytansinoid.^[46]^ Upon antigen binding, the FRα-ADC complex was rapidly internalized, leading to the release of DM4. DM4 exerts strong antimitotic activity by inhibiting microtubule dynamics,^[47]^ resulting in cell cycle arrest and apoptosis. Notably, MIRV enables active DM4 metabolites to diffuse from antigen-positive tumor cells to neighboring cells, effectively inducing a “bystander kill” effect.^[48]^ In our network analysis, mirvetuximab did not demonstrate a significant improvement in PFS, but exhibited a superior objective response rate (OR = 0.46; 95% CI: 0.23–0.85) compared to chemotherapy. Recently, 2 clinical trials reported the safety and efficacy of the combination of mirvetuximab soravtansine and bevacizumab in patients with PRrOC, showing improved overall response rates.^[49,50]^ Consequently, the Food and Drug Administration approved its use in adult patients with FRα-positive, platinum-resistant epithelial ovarian, fallopian tube, or primary peritoneal cancers on November 14, 2022. Therefore, it may serve as an alternative treatment option for patients with PRrOC who are positive for FRα.

Regarding the observed SAEs in the heatmap plot of this network analysis, nivolumab, mirvetuximab, and avelumab ranked as the top 3 regimens with fewer SAEs. The safety profile of nivolumab in this study aligns with systematic reviews and other studies, indicating that nivolumab is a relatively safe antitumor drug, with an overall incidence of SAEs of 11.2%.^[51]^ Conversely, bevacizumab plus chemotherapy ranked last in the heatmap, indicating a higher risk of SAEs than the other regimens investigated. This difference could be attributed to the pharmacological characteristics of anti-angiogenic agents that target vascular endothelial growth factor receptor (VEGF). These agents may inhibit endothelial NO synthase (eNOS), leading to reduced nitric oxide (NO) production, oxidative stress, activation of the endothelin system, and rarefaction, resulting in significant vascular toxicity, particularly hypertension and thromboembolism.^[52]^ Therefore, we recommend that oncologists carefully balance the risks of potentially serious toxicities against clinical benefits when considering strategies involving a combination of angiogenesis inhibitors in patients with PRrOC.

The subgroup analysis of hematologic SAEs, including neutropenia, thrombocytopenia, and anemia, is presented in a heatmap plot. The results indicate that lurbinectedin ranks lower than the other regimens of interest regarding hematologic SAEs. These findings are consistent with the original literature and align with the conclusions of a review article.^[53]^ Regarding non-hematological SAEs such as hypertension and gastrointestinal perforation, the risk of hypertension demonstrated similar results to a previous meta-analysis (RR = 6.57; 95% CI: 1.50–28.7).^[54]^

Our study had several strengths, primarily focusing on including phase III clinical trials, which helped reduce selection bias and inconsistencies among the included trials. However, it is important to acknowledge certain limitations in the selection of phase III RCTs, which may have resulted in an underestimation of the PRrOC findings. Moreover, the moderate heterogeneity observed in this study could be attributed to variations in the study design and the combination of different chemotherapeutic drugs. Nevertheless, the robustness of our sensitivity analysis helps to mitigate the impact of this limitation.

## 5. Conclusions

In conclusion, combining bevacizumab with chemotherapy or lurbinectedin may offer superior PFS improvement compared to other treatments. However, it is important to note that certain regimens are associated with a higher risk of SAEs. Therefore, oncologists should carefully weigh the benefits and risks when selecting a specific treatment option for individual patients. Furthermore, it is essential to consider the existing evidence regarding the combination of immunomodulatory agents with other immunomodulators, PARP inhibitors, and cytotoxic chemotherapy strategies for PRrOC. Future research efforts should focus on enhancing our understanding of disease biology and identifying predictive biomarkers of responses to immunotherapies or novel drug targets. Additionally, appropriate and effective symptom management plans need to be developed to improve patients’ quality of life.^[55]^

## Author contributions

**Data curation:** Hei-Tung Yip.

**Investigation:** Henry W. C. Leung.

**Methodology:** Agnes L. F. Chan.

**Software:** Agnes L. F. Chan.

**Writing – original draft:** John Hang Leung.

**Writing – review & editing:** Shyh-Yau Wang.

## Supplementary Material








