# Supplementary material for: Comparison of target agent treatment strategies for platinum-resistant recurrent ovarian cancer: A Bayesian network meta-analysis
Source: Medicine (Baltimore). 2024 May 24;103(21):e38183. doi: 10.1097/MD.0000000000038183 (PMC11124750; doi:10.1097/MD.0000000000038183)
Supplement: Supplementary file 1 [file medi-103-e38183-s001.pdf]

SUPPLEMENTARY SEARCH INFORMATION

Database: Pubmed

Search date: 10/31/2021

PATIENTS (Platinum-resistant or refractory ovarian cancer)

1. Platinum-resistant or refractory ovary cancer **(2790 papers)**
2. Randomized controlled trial **(742,654 papers)**
3. (Platinum-resistant or refractory ovary cancer) AND (Randomized controlled trial) **(178 papers)**

INTERVENTIONS/COMPARISONS

4. Bevacizumab **(20,561 papers)**
5. Chemotherapy **(3,682,829 papers**)
6. Bevacizumab AND Chemotherapy（**16,846 paper**）

((Platinum-resistant or refractory ovary cancer) AND (Randomized controlled trial OR Phase III)) AND ((Chemotherapy) AND (Bevacizumab)) **(46 paper)**

7. Pertuzumab **(1379 paper)**

(pertuzumab) AND ((Platinum-resistant or refractory ovary cancer) AND (Randomized controlled trial)) **(7 paper)**

8. Avelumab **(2104 paper)**

(Avelumab) AND ((Platinum-resistant or refractory ovary cancer) AND (Randomized controlled trial)) **(4 paper)**

9. anti-angiogenetic agents **(81,434 paper)**

(anti-angiogenetic agents) AND ((Platinum-resistant or refractory ovary cancer) AND (Randomized controlled trial OR Phase III trials)) **(40 paper)**

10. target therapy **(730,941 paper)**

('target therapy') AND ('platinum resistant' OR 'refractory ovary cancer') AND ('randomized controlled trial' OR Phase III) **(176 paper)**

Database: EMBASE
Search date:11/3/2021
PATIENTS (advanced or metastatic breast cancer)
Search for title or abstract
1.Platinum-resistant or refractory ovary cance**r (12665 papers**)
2.Randomized controlled trial **(997156 papers)**
platinum resistance' OR (('platinum'/exp OR platinum) AND ('resistance'/exp OR resistance)) OR 'refractory ovary ca' OR (refractory AND ('ovary'/exp OR ovary) AND ('ca'/exp OR ca))
(12665 paper)

INTERVENTIONS/COMPARISONS
1. Bevacizumab **(33 papers)**
('platinum resistant' OR 'refractory ovary cancer' OR (refractory AND ('ovary'/exp OR ovary) AND ('cancer'/exp OR cancer))) AND bevacizumab:ab,ti AND 'randomized controlled trial':ab,ti **(2 papers)**
2. Pertuzumab **(6325papers)**
('platinum resistant' OR 'refractory ovary cancer' OR (refractory AND ('ovary'/exp OR ovary) AND ('cancer'/exp OR cancer))) AND pertuzumab :ab,ti AND 'randomized controlled trial':ab,ti **(6 papers)**
3. Target therapy **(423,094 papers)**
('target therapy' OR (('target'/exp OR target) AND ('therapy'/exp OR therapy))) AND ('platinum resistant':ab,ti OR 'refractory ovary cancer':ab,ti) AND 'randomized controlled trial':ab,ti **(1 papers)**
4. Antiangiogenetic agent **(0 paper)**
'antiangiogenetic agent' OR (antiangiogenetic AND ('agent'/exp OR agent))) AND ('platinum resistant':ab,ti OR 'refractory ovary cancer':ab,ti) AND 'randomized controlled trial':ab,ti **(0 paper)**

Database: Cochrane Central Register of Controlled Trials
Search date: :11/3/2021

PATIENTS (advanced or metastatic breast cancer)
Search for title or abstract

**1.**	Platinum-resistant or refractory ovary cancer [TI, AB, KY]	**(591 papers)**
**2.**	Phase III randomized controlled trial [TI, AB, KY]	**(51186 papers)**

INTERVENTIONS/COMPARISONS

**3**	**Bevacizumab and combined chemotherapy [TI, AB, KY]**	**(1036 papers)**
**4**	**#1 AND #2 AND #3**	**(15 papers)**
**5**	**Pertuzumab**	**(577 papers)**
**6**	**#1 AND #2 AND #5**	**(9 papers)**
**7**	**antiangiogenetic agent**	**(6 papers)**
**8**	**#1 AND #2 AND #7**	**(0 paper)**
**9**	**target therapy**	**(21708 papers)**
**10**	**#1 AND #2 AND #9**	**(5 papers)**

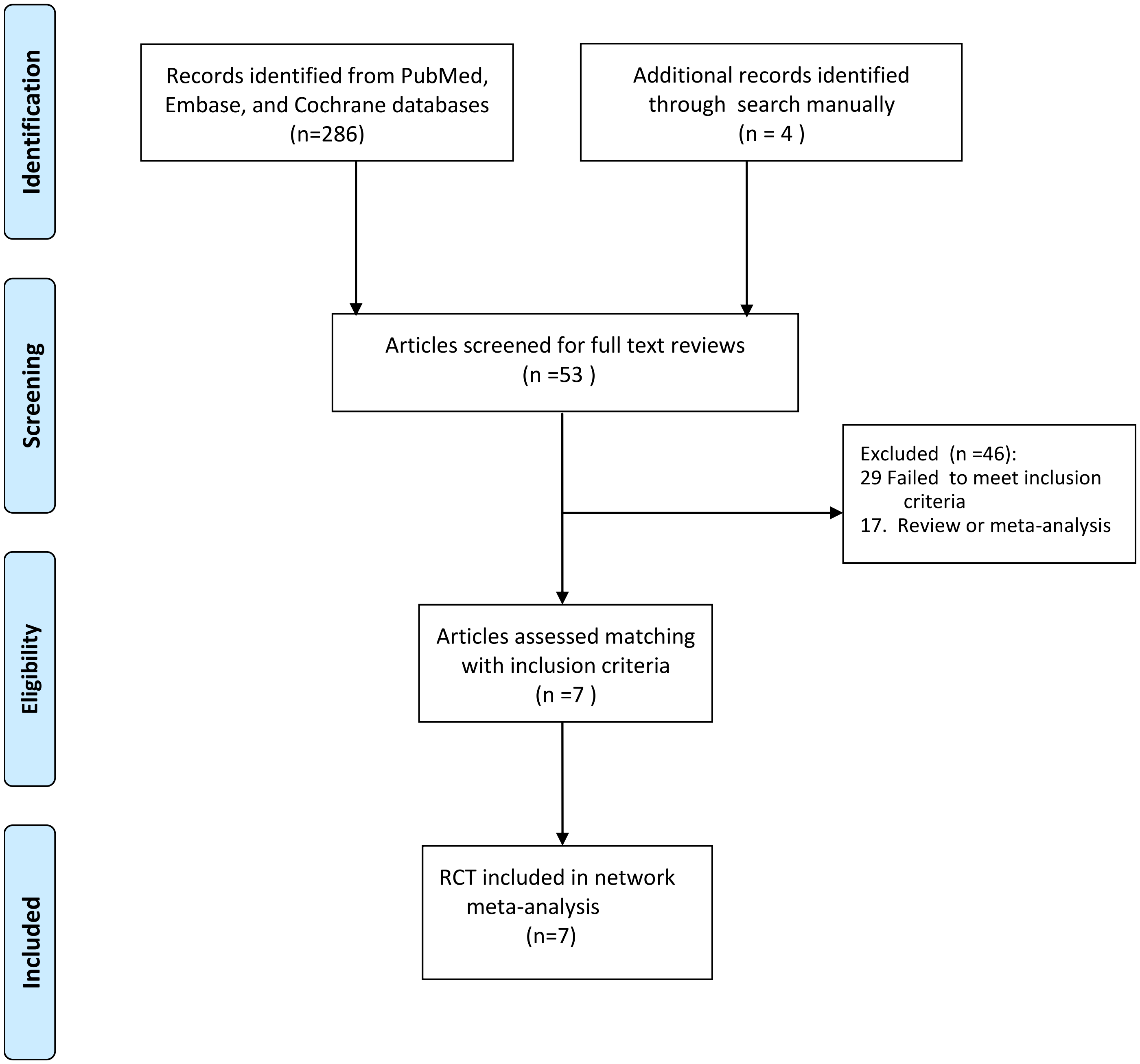

Supplementary ¯Figure 1. The flow chart summarizing the process for the identification of the eligible randomized controlled trials

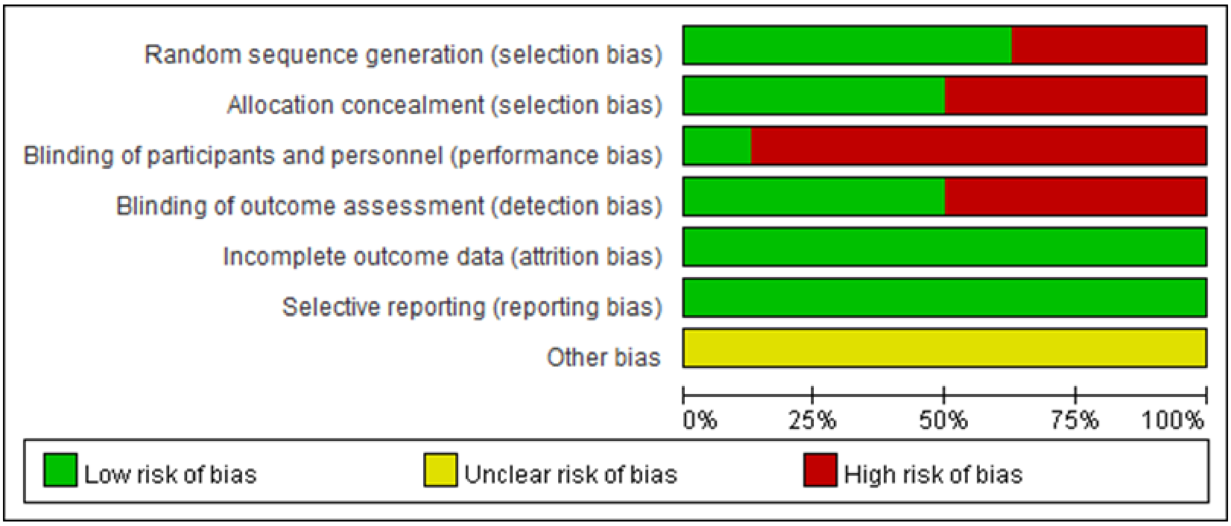

Supplementary Figure 3. Risk of bias table in direct comparison (green: low risk of bias; red: high risk of bias; yellow; unclear risk of bias).

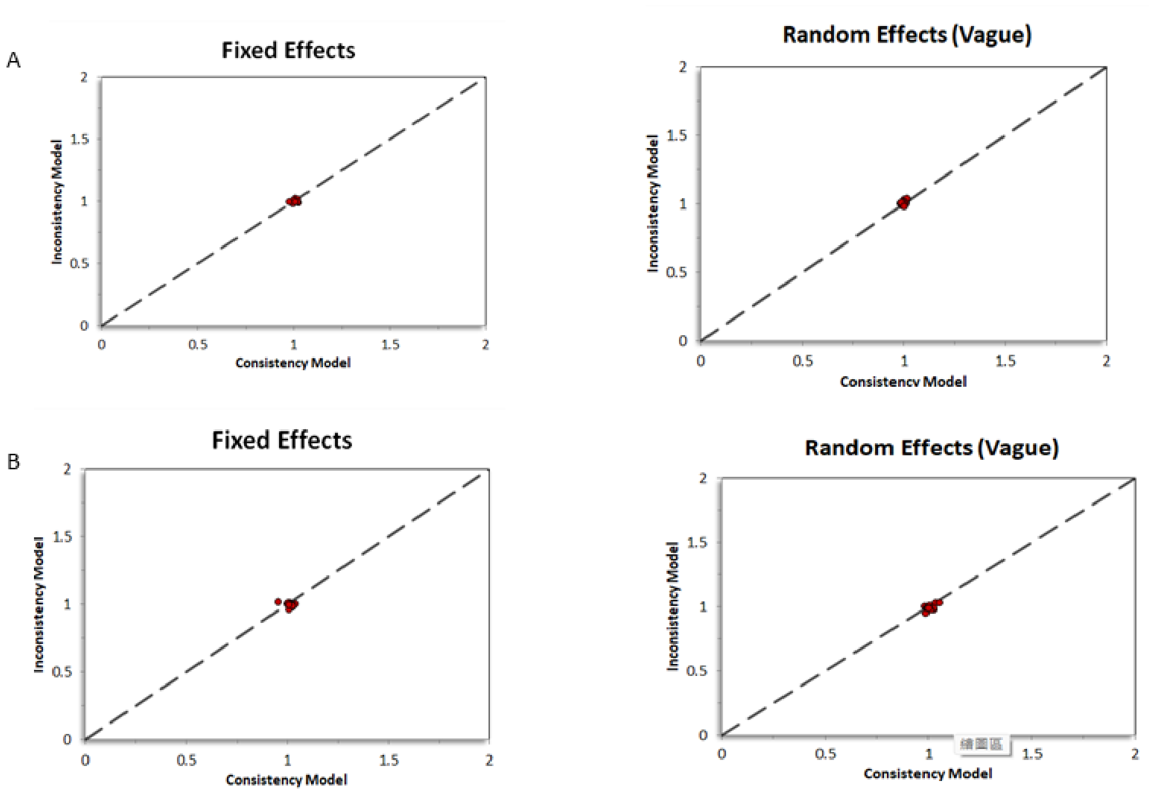

Supplementary Figure 3. Inconsistency plot for efficacy in terms of (A) Overall survival (B) Progression free survival of target therapies and immunotherapies. Plot of individual data points for the consistency model (horizonal axis) and the inconsistency model (vertical axis), along with the equality line.
