# Supplementary material for: Comparison of target agent treatment strategies for platinum-resistant recurrent ovarian cancer: A Bayesian network meta-analysis
Source: Medicine (Baltimore). 2024 May 24;103(21):e38183. doi: 10.1097/MD.0000000000038183 (PMC11124750; doi:10.1097/MD.0000000000038183)
Supplement: Supplementary file 2 [file medi-103-e38183-s002.doc]

Articles screened for full text reviews

(n =53 )

**Screening**

**Included**

**Eligibility**

Excluded (n =46):

29 Failed to meet inclusion criteria

17. Review or meta-analysis

Articles assessed matching with inclusion criteria

(n =7 )

RCT included in network

meta-analysis

(n=7)

**Identification**

Supplementary ˉFigure 1. The flow chart summarizing the process for the identification of the eligible randomized controlled trials

Records identified from PubMed,

Embase, and Cochrane databases (n=286)
(n =177 )

Additional records identified through search manually
(n = 4 )
