# Supplementary material for: Comparison of target agent treatment strategies for platinum-resistant recurrent ovarian cancer: A Bayesian network meta-analysis
Source: Medicine (Baltimore). 2024 May 24;103(21):e38183. doi: 10.1097/MD.0000000000038183 (PMC11124750; doi:10.1097/MD.0000000000038183)
Supplement: Supplementary file 3 [file medi-103-e38183-s003.pptx]

## Slide 1
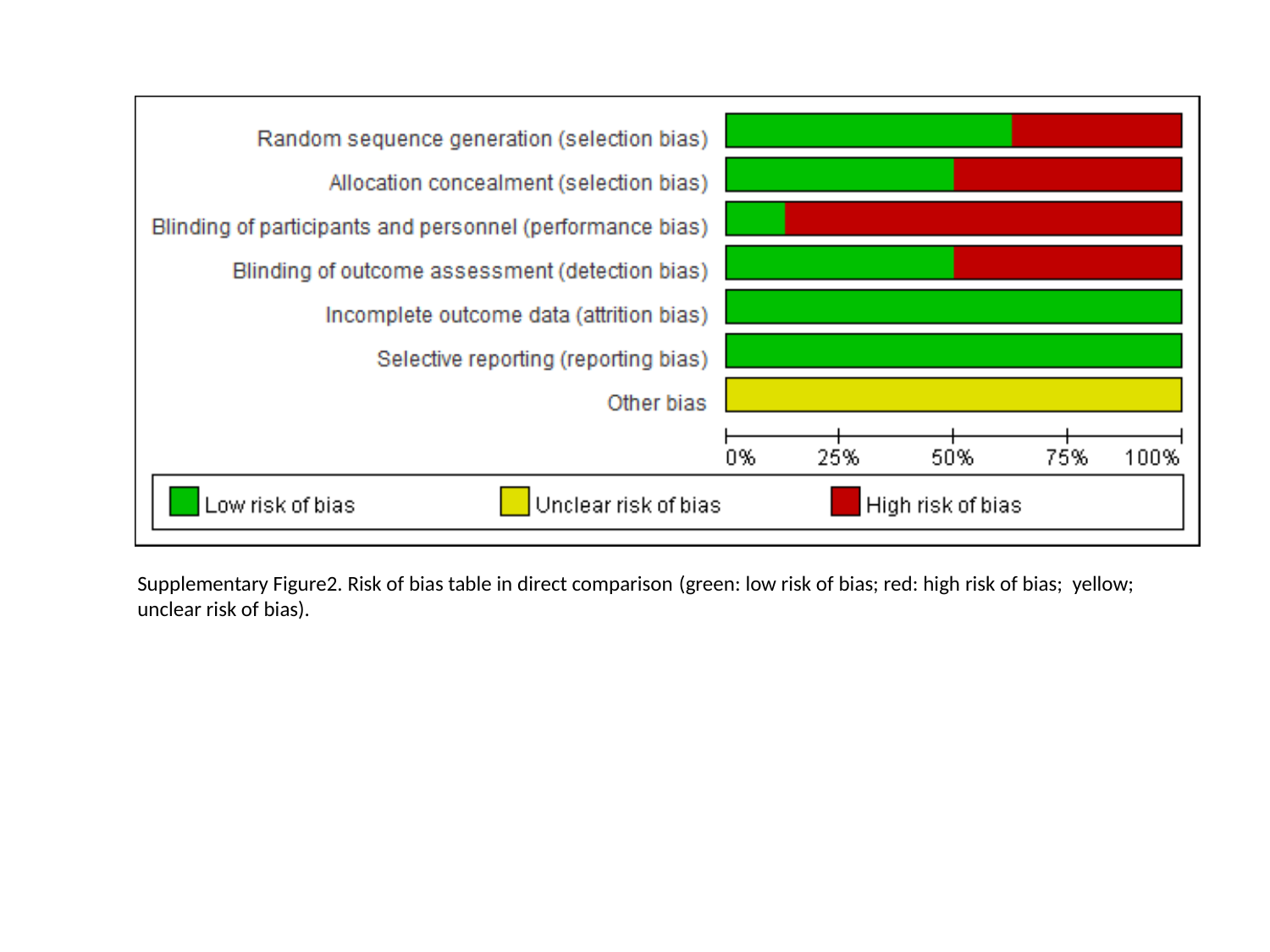

Supplementary Figure2. Risk of bias table in direct comparison (green: low risk of bias; red: high risk of bias; yellow; unclear risk of bias).
