# Supplementary material for: Comparison of target agent treatment strategies for platinum-resistant recurrent ovarian cancer: A Bayesian network meta-analysis
Source: Medicine (Baltimore). 2024 May 24;103(21):e38183. doi: 10.1097/MD.0000000000038183 (PMC11124750; doi:10.1097/MD.0000000000038183)
Supplement: Supplementary file 4 [file medi-103-e38183-s004.pptx]

## Slide 1
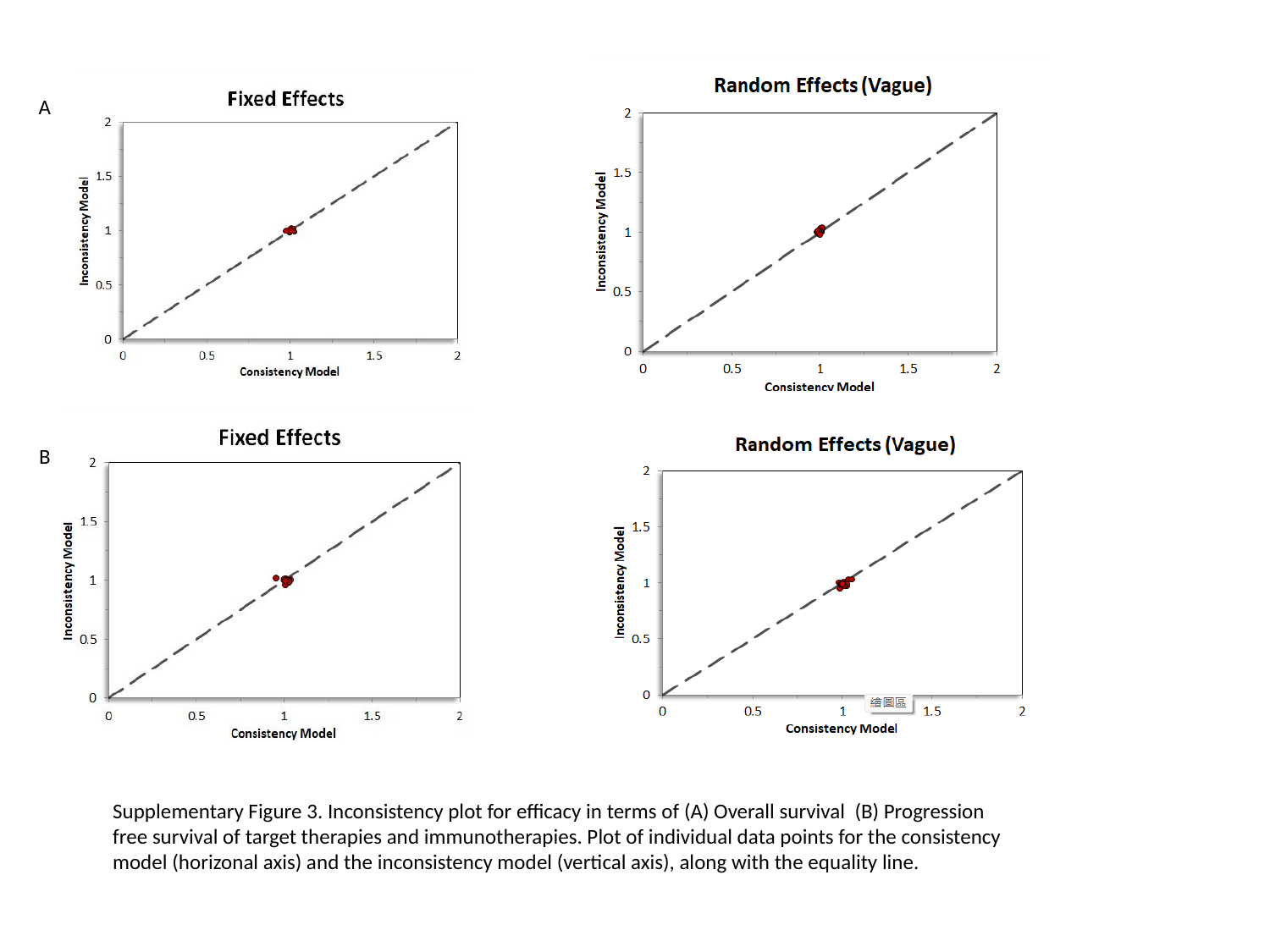

A
B
Supplementary Figure 3. Inconsistency plot for efficacy in terms of (A) Overall survival (B) Progression free survival of target therapies and immunotherapies. Plot of individual data points for the consistency model (horizonal axis) and the inconsistency model (vertical axis), along with the equality line.
